# Mesenchymal stem cell-mediated delivery boosts the efficacy of suicide gene therapy based on retroviral replicating vectors in peritoneally disseminated cancer

**DOI:** 10.1038/s41417-026-01047-2

**Published:** 2026-06-13

**Authors:** Shuji Kubo, Yuya Takeuchi, Emiko Sonoda-Fukuda, Nao Ogawa, Shugo Shinoda, Akiko Nakano-Doi, Takayuki Nakagomi, Soichiro Funaki, Noriyuki Kasahara

**Affiliations:** 1 Laboratory of Molecular and Genetic Therapeutics, Institute for Advanced Medical Sciences, Hyogo Medical University, Nishinomiya, Hyogo Japan; 2 Department of Biomedical Chemistry, School of Science and Technology, Kwansei Gakuin University, Sanda, Hyogo Japan; 3 Department of Biological Sciences, School of Biological and Environmental Sciences, Kwansei Gakuin University, Sanda, Hyogo Japan; 4 Laboratory of Neural Repair and Regeneration, Institute for Advanced Medical Sciences, Hyogo Medical University, Nishinomiya, Hyogo Japan; 5 Division of Thoracic Surgery, Department of Surgery, Hyogo Medical University, Nishinomiya, Hyogo Japan; 6 Departments of Neurological Surgery and Radiation Oncology, University of California, San Francisco, CA USA

**Keywords:** Mesothelioma, Genetic vectors

## Abstract

Retroviral replicating vectors (RRVs) hold promise for cancer gene therapy but face limitations due to their relatively low titers and susceptibility to inactivation by body fluids, limiting their application to intratumoral delivery. To overcome these challenges and target metastatic cancers, we investigated the use of tumor-homing mesenchymal stem cells (MSCs) as RRV carriers in a clinically relevant model of malignant peritoneal mesothelioma. MSCs derived from adipose tissue, bone marrow, and umbilical cord demonstrated significant migration toward mesothelioma cells and were permissive to RRV infection and production. MSCs transferred RRVs to tumor cells more effectively in direct co-culture than in Transwell assays. Using peritoneally disseminated cancer models, we confirmed that MSCs enhanced RRV transfer, even under ascites-mimicking conditions. In vivo biomolecular imaging and flow cytometry revealed markedly reduced RRV transduction under ascites conditions; however, MSC/RRV delivery significantly enhanced intratumoral viral transmission. Furthermore, while both direct RRV and MSC/RRV treatments were effective in non-ascites models, MSC/RRV delivery achieved superior antitumor efficacy in ascites-mimicking models, resulting in robust tumor suppression and prolonged survival. These findings highlight the potential of MSCs to overcome the limitations of RRV and suggest that MSC-based RRV-mediated suicide gene therapy represents a promising strategy for peritoneally disseminated cancers complicated by cancerous ascites.

## Introduction

Retroviral replicating vectors (RRVs) derived from amphotropic murine leukemia virus (AMLV) exhibit unique characteristics that enable highly efficient and tumor-selective gene transfer [1]. This selectivity arises from their inability to infect non-dividing cells and the defective antiviral defenses commonly found in cancer cells and immunoprivileged tumor environments [1]. Therefore, RRVs undergo multiple rounds of replication and spread throughout the tumor, increasing the overall proportion of infected cells and infected areas. Unlike other viruses used in cancer virotherapy, RRVs are non-cytolytic but can be engineered to carry suicide genes that mediate synchronized killing of infected tumor cells upon prodrug administration. For example, RRVs engineered to express the yeast cytosine deaminase (CD) gene convert the prodrug 5-fluorocytosine (5-FC) into the toxic metabolite 5-fluorouracil. This approach has demonstrated high efficiency in killing various cancer cells in vitro and in vivo following the administration 5-FC [2–6]. In other words, RRV-mediated suicide gene therapy is the ultimate intracellular chemotherapy that is largely confined to tumor cells and minimizes systemic side effects. This therapy is further characterized by its long-term tumor control capabilities. RRVs persist once integrated into the chromosomes of infected cancer cells, allowing continuous CD expression in the original and daughter tumor cells. Consequently, RRVs are continually produced within the tumor, enabling repeated prodrug activation and sustained tumor suppression [5, 7].

The clinical development of RRV-based suicide gene therapy has advanced substantially, supported by extensive preclinical studies and multiple early-phase clinical trials conducted in the United States and Europe. Direct intratumoral administration of RRVs has been evaluated in patients with malignant gliomas, demonstrating an acceptable safety profile as well as biological and antitumor activity, including evidence of tumor control in subsets of patients [1, 7–9]. Although a subsequent international phase II/III trial did not meet its primary endpoint of overall survival in the overall study population, exploratory analyses suggested potential benefit in selected patient subgroups, supporting continued investigation of this therapeutic platform [1, 8].

RRV-mediated suicide gene therapy has also demonstrated efficacy against a variety of cancers, including glioma [6], mesothelioma [3, 4], lung cancer [9], gastric cancer [10], pancreatic cancer [11], ovarian cancer [12], and osteosarcoma [13]. In malignant mesothelioma, previous studies have shown high expression of RRV entry receptors and efficient viral transmission in tumor cells, resulting in significant therapeutic efficacy following intratumoral RRV administration in subcutaneous tumor models [3, 4]. In contrast, intraperitoneal administration of RRVs in peritoneal dissemination models, characterized by widely scattered small tumor nodules, has shown limited infection efficiency and antitumor effects, particularly under ascites-mimicking conditions (unpublished observations and results presented in this study). Under such conditions, RRVs are diluted within the peritoneal fluid and are susceptible to inactivation by complement and antibodies, as is generally observed for retroviruses [14, 15]. Consequently, direct intraperitoneal RRV administration is considered suboptimal for the treatment of peritoneally disseminated cancers complicated by ascites. To address this limitation, a method for efficiently delivering RRVs to tumors must be developed.

One promising approach involves using mesenchymal stem cells (MSCs) as cellular carriers for RRVs. MSCs derived from mesodermal tissues, including bone marrow, umbilical cord, and adipose tissue, exhibit tumor-homing properties, contribute to tumor stroma formation, and possess low immunogenicity due to limited activation of host immune responses [16–19]. These features make MSCs attractive vehicles for delivering therapeutic agents, including genes and viral vectors, in cancer therapy [20]. Indeed, enhanced antitumor efficacy has been reported for MSC-mediated delivery of oncolytic viruses, such as measles virus [21], adenovirus [22–24], and herpes virus [25], supporting the potential of this strategy to advance cancer virotherapy.

In this study, we investigated the feasibility and therapeutic efficacy of using tumor-homing MSCs as RRV carriers in models of peritoneally disseminated cancer. We demonstrated that MSCs can effectively deliver RRVs to tumor sites, achieve efficient tumor transduction, and enhance the antitumor efficacy of RRV suicide gene therapy, particularly under ascites-mimicking conditions.

## Materials and methods

### Cell lines

Human MSCs derived from adipose tissue (MSC-ad), bone marrow (MSC-bm), and umbilical cord (HUMSC, hereafter referred to as MSC-uc) were purchased from ScienCell Research Laboratories (Carlsbad, CA, USA) and maintained in mesenchymal stem cell medium (ScienCell Research Laboratories). The human mesothelioma cell line MSTO-211H was obtained from the American Type Culture Collection (Manassas, VA, USA) and cultured in Roswell Park Memorial Institute 1640 (RPMI 1640) medium (Nacalai Tesque, Kyoto, Japan) supplemented with 10% fetal bovine serum (FBS; Atlas Biologicals, Fort Collins, CO, USA). Normal human adult mesothelial cells (NMCs) were obtained from Zen-Bio, Inc. (Research Triangle Park, NC, USA) and cultured in mesothelial cell growth medium (Zen-Bio, Inc.). Human embryonic kidney 293T cell lines were maintained in Dulbecco’s modified Eagle’s medium (DMEM; Nacalai Tesque, Kyoto, Japan) supplemented with 10% FBS. All cells were cultured at 37 °C in a humidified atmosphere containing 5% CO₂.

### Vector plasmids and virus production

As previously reported [3, 4], the RRV vector plasmid contained a full-length replication-competent amphotropic murine leukemia virus genome, including gag polyprotein, reverse transcriptase, integrase, and viral envelope genes, followed by an internal ribosome entry site and sequences encoding green fluorescent protein (pRRV-GFP), CD (pRRV-CD), or firefly luciferase/Venus fusion protein [26] (pRRV-fLuc/Venus). For virus production, 293T cells were used as packaging cells. The culture medium was replaced with serum-free DMEM at the time of transfection, and the cells were transiently transfected with the vector plasmid using Lipofectamine 2000 (Life Technologies Japan, Tokyo, Japan). The cells were then maintained in serum-free DMEM for 48 h. Virus-containing supernatants were collected, filtered through a 0.45-µm filter, and stored at −80 °C until use.

A self-inactivating lentiviral vector expressing mCherry (LVSKc-mCherry) was created by replacing the GFP sequence in sinSKcmv-EGFP (LV-GFP) with mCherry cDNA, as previously described [3, 27]. The virus preparations were produced by transient cotransfection of 293T cells as described previously [3, 28]. Virus titers were determined by measuring fluorescent protein expression in MSTO-211H cells using a FACSCalibur flow cytometer (Becton Dickinson Japan, Tokyo, Japan) and expressed as transducing units (TU) per milliliter.

### Cell labeling with mCherry fluorescence

For fluorescent labeling, MSTO-211H cells and human MSCs were cultured in complete growth medium to 30–50% confluency in 12-well plates and transduced with the lentiviral vector LVSKc-mCherry. The cells were infected at a multiplicity of infection of 10 for MSTO-211H cells and 100 for MSCs and incubated for 72 h post-transduction. After transduction, cells were passaged, and clones exhibiting high mCherry fluorescence were isolated by limiting dilution in 96-well plates to establish stable MSTO-211H-mCherry and MSC-mCherry cell lines. High-fluorescence clones were expanded and maintained under standard culture conditions. mCherry expression was confirmed using flow cytometry, and only cell populations with ≥99% mCherry-positive cells were used for subsequent experiments.

### In vitro migration assay

MSTO-211H tumor cells (1 × 10^5^ cells) were seeded in the bottom wells of 24-well plates. The following day, Transwell inserts with 8 µm-pore filters were placed into the wells. Both the upper and lower chambers were filled with RPMI 1640 supplemented with 10% FBS. mCherry-labeled human MSCs (2 × 10⁴ cells/well; MSC-ad, MSC-bm, and MSC-uc), suspended in RPMI 1640 containing 10% FBS, were added to the upper chamber. After 3 days of incubation, non-migrated cells on the upper surface of the filter were removed using a cotton swab. Migrated cells on the underside of the filter were stained with crystal violet and counted manually under a microscope at ×200 magnification in three randomly selected fields per insert. The mean value was used for subsequent analyses. In addition, cells in the lower chamber, consisting of migrated mCherry-labeled MSCs and pre-existing unlabeled tumor cells, were also analyzed for mCherry expression using flow cytometry.

### Transduction of MSCs with RRV

For the RRV infectivity assay, MSC-ad, MSC-bm, and MSC-uc (2 × 10^5^ cells/well) were seeded in 6-well plates and inoculated with varying volumes (0.1, 1, and 10 µL) of RRV-GFP. MSTO-211H cells were used as a positive control [3, 4], and 293T cells served as a low-infectivity control. GFP expression was analyzed using flow cytometry on day 2 post-infection to determine the RRV titer in each cell type.

For the RRV production assay, MSC-ad, MSC-bm, and MSC-uc (2 × 10^5^ cells/well in 6-well plates) were inoculated with 100 µL of RRV-GFP and passaged three times. MSTO-211H cells were used as a positive control [3, 4], whereas NMCs and 293T cells served as a low-RRV-production controls. Virus-containing supernatants were subsequently collected and used to infect MSTO-211H tumor cells. GFP expression was evaluated by flow cytometry on day 2 post-infection to assess the RRV production efficiency.

To generate fully RRV-infected MSC populations for subsequent experiments, MSCs (1 × 10^4^ cells) in a 24-well plate were infected three times at 1-day intervals with 100 μL of RRV-GFP, RRV-fLuc/Venus, or RRV-CD. The cells were then re-seeded, allowed to proliferate, and aliquoted for cryopreservation.

### In vitro transmission of RRV from MSCs to tumor cells

MSTO-211H tumor cells (1 × 10^5^ cells) were seeded in the bottom wells of a 24-well plate. Transwell inserts with 0.4 µm-pore filters were placed in the wells the following day, and MSCs fully infected with RRV-GFP (5 × 10⁴ cells/well) were added to the upper chamber. After 3 days, MSTO-211H cells were harvested, and GFP expression was analyzed using fluorescence microscopy and flow cytometry.

For co-culture experiments, MSTO-211H/mCherry cells (5 × 10⁵ cells) and MSC/RRV-GFP cells (5 × 10⁴ cells) were mixed at a 10:1 ratio and cultured in 6-well plates for 3 days. Fluorescent protein expression was analyzed using microscopy and flow cytometry.

### Tumor cell-killing effect of MSC-mediated RRV-mediated suicide gene therapy

MSTO-211H cells were mixed with MSC/RRV-CD cells at various ratios (0–8% MSC) and cultured in 6-well plates for 7 days. The cells were then transferred to 96-well plates and treated with different concentrations of 5-FC (Tokyo Chemical Industry, Tokyo, Japan) for 6 days. Cell viability of triplicate cultures was assessed using the alamarBlue assay (Alamar Biosciences, Inc., Sacramento, CA) according to the manufacturer’s protocol. Fluorescence was measured using an ARVO X4 multilabel plate reader (PerkinElmer Japan, Tokyo, Japan) with excitation and emission at 544 and 590 nm, respectively. Viability was calculated by normalizing fluorescence values to untreated controls for each MSC proportion.

### In vivo transmission of RRV from MSC to intraperitoneal tumor xenografts

Female BALB/c-nu/nu (nude) mice (CLEA Japan, Tokyo, Japan) were maintained under specific-pathogen-free conditions, and all procedures were approved by the Animal Research Committee of Hyogo Medical University. Intraperitoneal tumor xenografts were established by injecting MSTO-211H/mCherry cells (2 × 10⁶ cells suspended in 500 µL of phosphate-buffered saline [PBS; Nacalai Tesque, Kyoto, Japan]) into 5-week-old mice. One week later, the mice (*n* = 5 per group) were randomly allocated to experimental groups using simple randomization without blinding and received intraperitoneal injections of either MSCs fully infected with RRV-fLuc/Venus (1 × 10⁶ cells suspended in 500 µL PBS) or RRV-fLuc/Venus alone (1 × 10⁶ TU in 500 µL PBS).

Four weeks later, mCherry and fLuc signals were analyzed using the IVIS Lumina II (Caliper Life Sciences Inc., Hopkinton, MA, USA), and mice in which tumors failed to establish were excluded from subsequent analyses. For in vivo imaging, D-luciferin (150 mg/kg body weight; Biosynth, Compton, UK) was administered intraperitoneally, and mice were anesthetized by isoflurane inhalation (Mylan Inc., Canonsburg, PA, USA). Regions of interest (ROIs) encompassing the entire intraperitoneal area were defined using Living Image software ver. 4.2 (Caliper Life Sciences). Bioluminescence signals were quantified as total photon flux (photons/s), and fluorescence signals as total radiant efficiency.　Identical ROI settings were applied to all animals within each experimental group.

After imaging, tumors were harvested, enzymatically dissociated into single-cell suspensions using Triple Enzyme Solution (Thermo Fisher Scientific, Waltham, MA, USA), and analyzed for Venus expression using flow cytometry to assess transduction efficiency within the tumors.

To establish ascites-mimicking models, mice (*n* = 5 per group) were inoculated intraperitoneally with MSTO-211H/mCherry cells (1 × 10⁶ cells suspended in 500 µL PBS) and subsequently received three intraperitoneal injections (days 0, 2, and 4) of 1 mL PBS containing 10% commercially available pooled human serum (Cosmo Bio Co., Ltd., Tokyo, Japan) following administration of either RRV-fLuc/Venus (2 × 10⁵ TU in 500 µL PBS) or MSC-ad/RRV-fLuc/Venus (2 × 10⁵ cells suspended in 500 µL PBS). The mCherry and fLuc signals were evaluated using the IVIS system 3 weeks later.

### RRV-mediated suicide gene therapy in a peritoneally disseminated tumor xenograft model

The peritoneally disseminated tumor xenograft model was established using the same procedure as that used in the in vivo RRV transmission experiments. Sample size was determined based on prior in vivo studies using RRVs, in which a group size of 10 animals was sufficient to detect biologically meaningful differences in tumor burden and survival outcomes.

In non-ascites models, mice bearing MSTO-211H/mCherry xenografts (*n* = 10 per group) received intraperitoneal injections of 500 µL of PBS, RRV-CD, MSC-ad, or MSC-ad fully infected with RRV-CD, as described for the in vivo RRV transmission experiments. In ascites-mimicking models, the same treatments were administered using PBS containing 10% pooled human serum. One week after tumor cell implantation, 5-FC was administered intraperitoneally at 500 mg/kg body weight in 500 μL PBS, three times per week throughout the experimental period. Tumor fluorescence intensities were quantified using IVIS and Living Image software.

### Statistical analysis

Except for survival data, statistical significance was assessed using Student’s *t* test and 1-way or 2-way analysis of variance (ANOVA), as appropriate. Data are presented as means ± standard deviations (SD). Survival data were analyzed using the Kaplan–Meier method. *P*-values of <0.01 were considered statistically significant in all analyses performed using GraphPad Prism 5 software (GraphPad Software, La Jolla, CA, USA).

## Results

### Transmigration of human MSCs to tumor cells

**Fig. 1:**
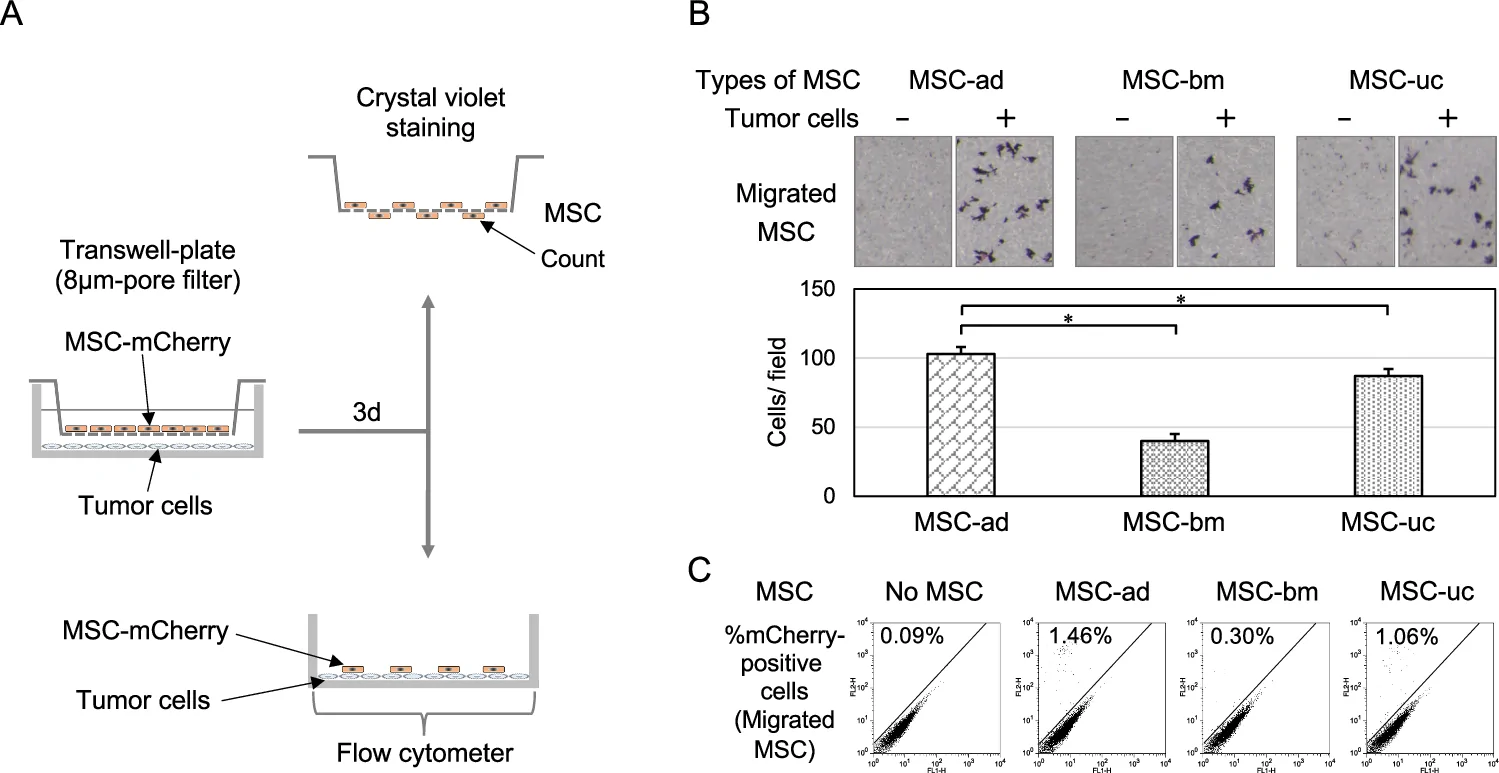
In vitro migration of MSCs toward tumor cells. **A** Experimental outline. The MSTO-211H tumor cells (1 × 10^5^ cells) were seeded into 24-well plates. The next day, Transwell inserts with 8 µm-pore filters were placed into the wells, and then human MSCs (2 × 10^4^ cells/well), labeled with mCherry (MSC-mCherry), derived from adipose tissue (MSC-ad), bone marrow (MSC-bm), and umbilical cord (MSC-uc), respectively, were added to the upper chamber. The inserts were removed 3 days later, and non-migrated cells on the upper side of the filters were carefully wiped off using cotton swabs. Migrated cells were analyzed on the underside of the filters (**B**) and on the lower chamber (**C**). **B** Microscopic analysis of anchorage-dependent MSC migration. Migrated MSCs on the underside of the filters were stained with crystal violet, photographed, and counted under a microscope. Representative microscopic images and number of migrated MSCs on the underside of the Transwell filters are shown. Data are shown as means ± SD from triplicate experiments (*p < 0.01). **C** Flow cytometric analysis of anchorage-independent MSC migration. Cells in the bottom well, which consisted of migrated mCherry-labeled MSCs and unlabeled tumor cells pre-existing in the lower chamber, were collected via trypsinization, and migrated MSCs (mCherry-positive) were analyzed using flow cytometry.

The tumor tropism of human MSCs toward mesothelioma cells was evaluated using a Transwell migration assay, with MSTO-211H human mesothelioma cells as the target. (Fig. 1A). All human MSC subtypes—derived from adipose tissue (MSC-ad), bone marrow (MSC-bm), and umbilical cord (MSC-uc)—exhibited significant transmigration toward tumor cells under both anchorage-dependent conditions, in which MSCs migrated toward tumor cells and adhered to the underside of the filter (Fig. 1B) and anchorage-independent conditions, in which MSCs migrated into and co-localized with tumor cells (Fig. 1C). Among these subtypes, MSC-ad demonstrated the highest migratory capacity.

### RRV infectivity and production capacity in MSCs

**Fig. 2:**
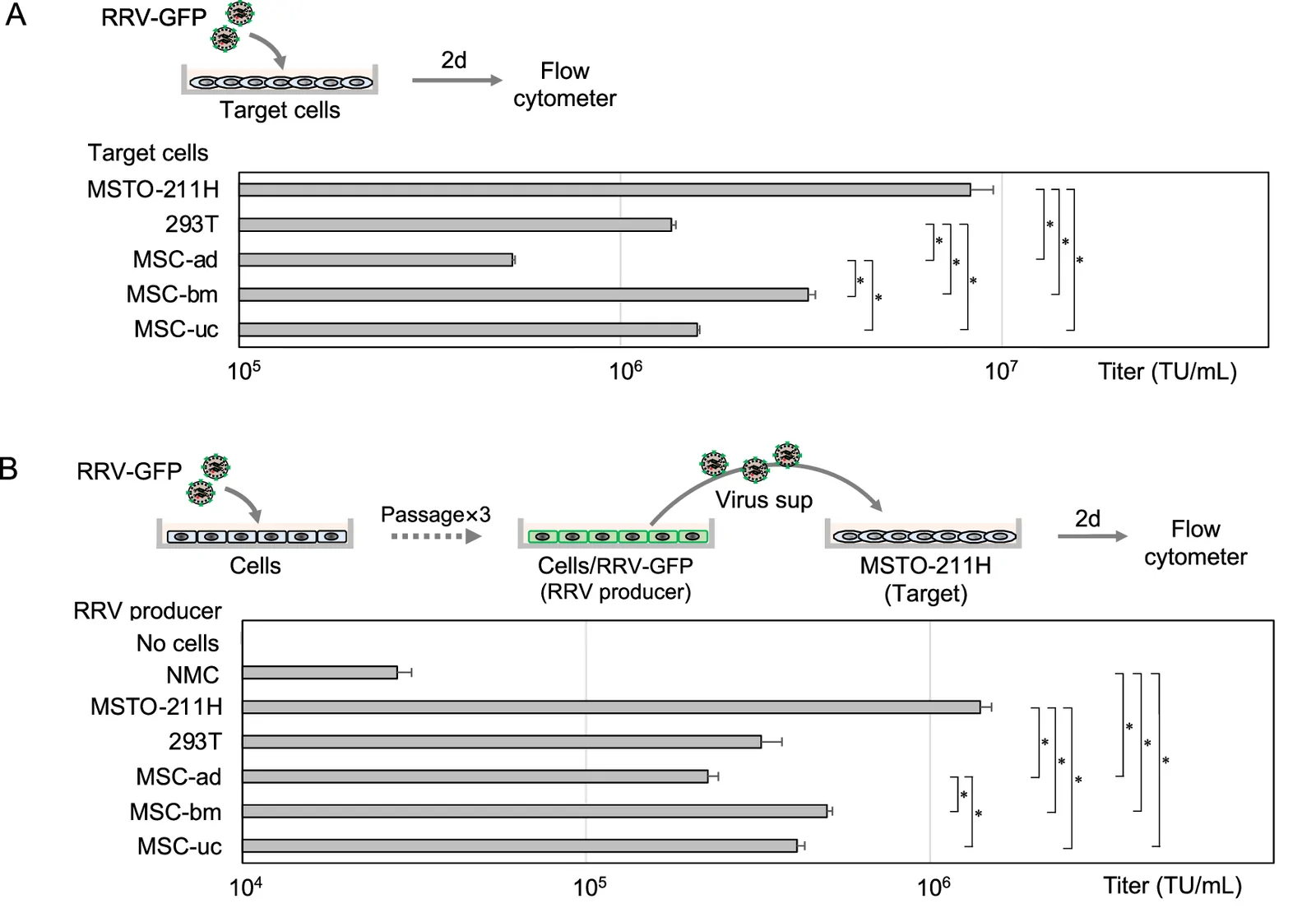
MSCs as RRV-producing cells. **A** RRV infectivity of MSCs. MSCs were infected with 10 µL of RRV-GFP. MSTO-211H cells were used as a positive control, and 293T cells served as a low-infectivity control. On day 2, GFP expression was evaluated using flow cytometry, and viral titers were determined. Data are presented as means ± SD from triplicate experiments (*p < 0.01). **B** RRV production potential of MSCs. Cells infected with 100 µL of RRV-GFP were passaged three times. The supernatants from MSC cultures were used to infect MSTO-211H tumor cells, and GFP expression was assessed using flow cytometry on day 2. The viral titers were determined for each cell type. Data are shown as means ± SD from triplicate experiments (*p < 0.01). NMC normal mesothelial cell.

The infectivity of RRVs in MSCs and their capacity to produce RRVs were evaluated. All MSC subtypes were susceptible to RRV infection, although the infectivity was significantly lower than that observed in MSTO-211H tumor cells (Fig. 2A). Among the MSCs, MSC-uc showed the highest infectivity, followed by MSC-bm, while MSC-ad exhibited the lowest infectivity, even below the level of normal 293T cells, which served as a low-infectivity control. Despite this low baseline infectivity, all MSC-ad cells became infected when exposed to high doses of RRV (data not shown).

To assess RRV production, supernatants were collected after three passages of fully infected MSCs and used to infect MSTO-211H cells. All MSC subtypes produced higher levels of RRVs than NMCs and showed RRV production comparable to that of 293T cells; however, their production levels remained lower than those observed in MSTO-211H cells (Fig. 2B). From a safety perspective, MSC-ad was considered a favorable carrier because of its combination of sufficient infectivity and relatively low RRV production, which may reduce premature viral dissemination before tumor localization.

### Transmission of RRV from MSCs to tumor cells

**Fig. 3:**
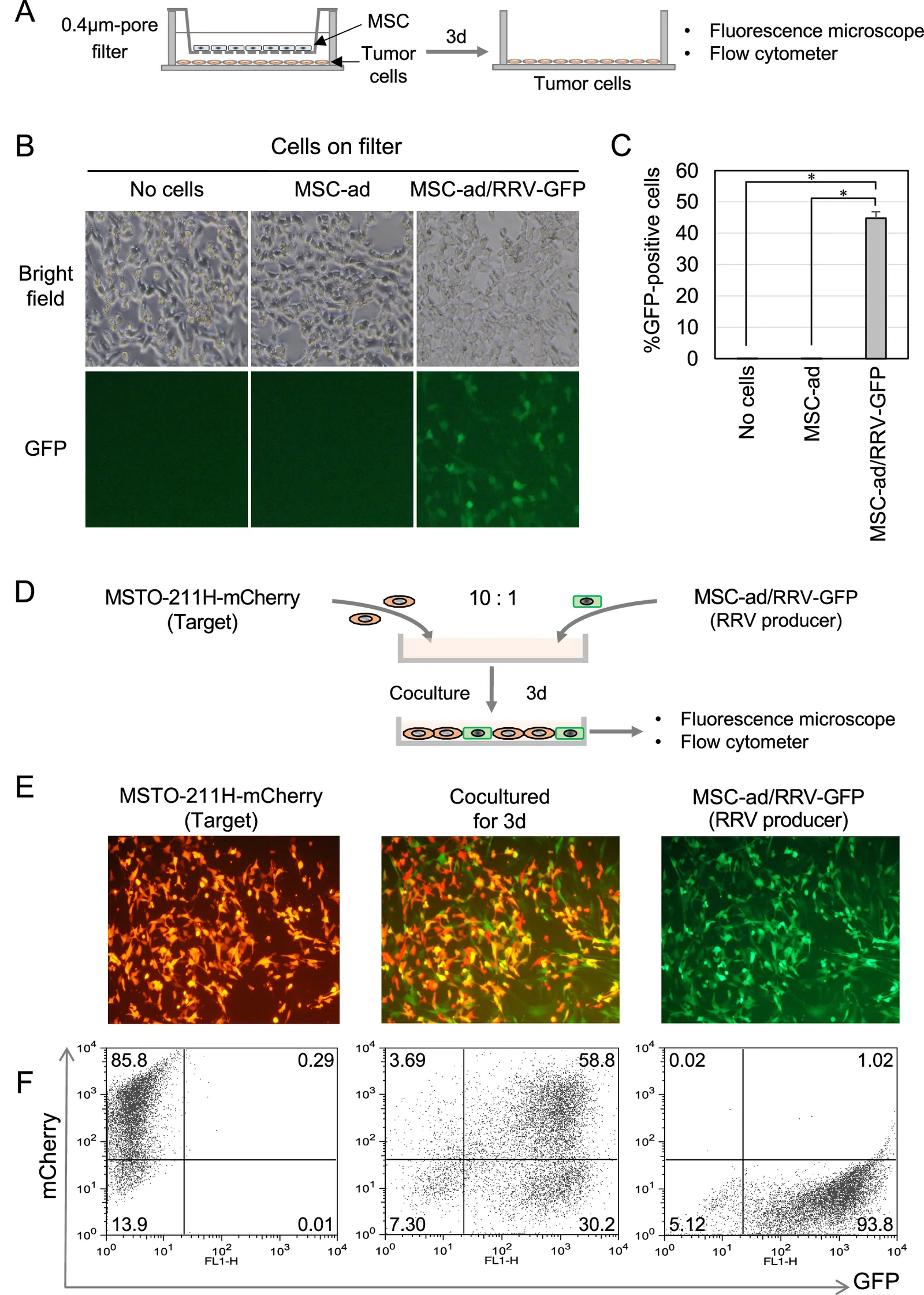
Transmission of RRV from MSC to tumor cells. **A** Transwell plate experiment outline. MSC-ad cells fully infected with RRV-GFP (MSC/RRV-GFP) were seeded into the upper chamber of Transwell inserts with 0.4 µm-pore filters, and MSTO-211H tumor cells were seeded into the lower chambers of 24-well plates. After 3 days, the tumor cells in the lower chambers were analyzed (**B** and **C**). **B** Fluorescence microscopy images. Representative bright-field and GFP fluorescence images of tumor cells are shown. **C** Flow cytometric analysis of GFP-positive tumor cells. The percentage of GFP-positive tumor cells was determined. Data are presented as means ± SD from triplicate experiments (*p < 0.01). **D** Coculture experiment outline. MSTO-211H tumor cells labeled with mCherry (MSTO-211H/mCherry) were cocultured with MSC/RRV-GFP at a 10:1 ratio for 3 days. Fluorescence expression was analyzed (**E** and **F**). **E** Fluorescence microscopy of the cocultured cells. Target tumor cells (left column, mCherry-positive) acquired GFP expression (middle column, mCherry and GFP-positive) after coculture with MSC-ad/RRV-GFP (right column, GFP-positive). **F** Flow cytometric analysis of fluorescence-positive tumor cells. Data are representative of multiple experiments conducted under varying conditions.

RRV transmission from MSCs to tumor cells was assessed using a Transwell assay, with MSC-ad and MSTO-211H tumor cells in the upper and lower chamber, respectively (Fig. 3A). After 3 days of incubation, GFP expression was observed in tumor cells, with an infection efficiency of 44.8 ± 2.1% (Fig. 3B, C). This result indicates that RRVs released from MSC-ad diffused through the filter and infected tumor cells in the lower chamber.

In contrast, when MSCs and tumor cells were directly cocultured (Fig. 3D), RRV transmission efficiency increased markedly. GFP- and mCherry-positive tumor cells accounted for 58.8% of the population, resulting in an estimated overall infection efficiency of 94.1% (calculated as 100 × 58.8/(3.69 + 58.8); Fig. 3E, F). These results indicate that direct cell-to-cell contact substantially enhances the transmission of RRV to target tumor cells.

### Tumor cell-killing by MSC-mediated RRV suicide gene therapy

**Fig. 4:**
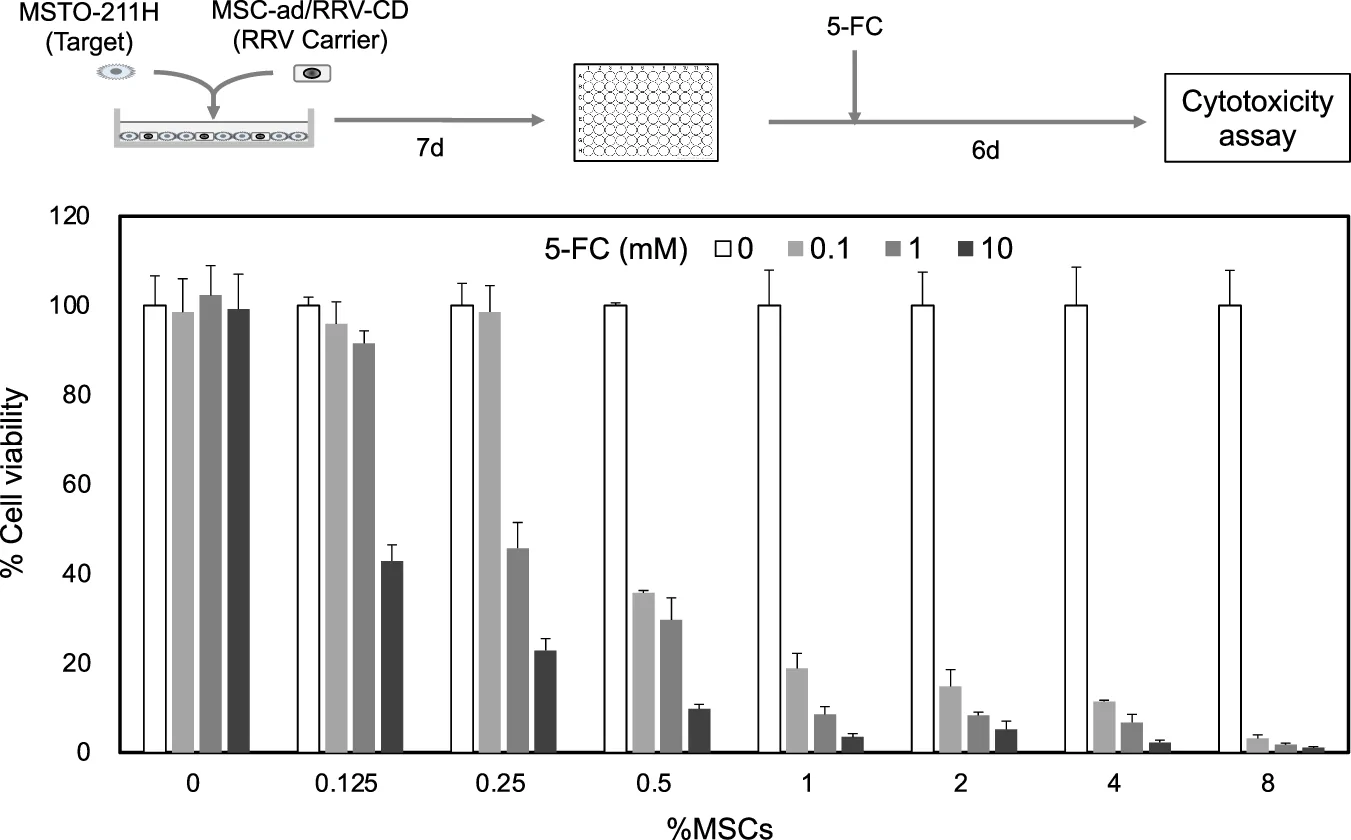
Tumor cell-killing by MSC-mediated RRV suicide gene therapy. MSTO-211H tumor cells were cocultured with MSC-ad/RRV-CD at varying ratios (0–8% MSC) in 6-well plates for 7 days. The cells were then transferred to 96-well plates, treated with various concentrations of 5-FC for 6 days, and assessed for viability using the alamarBlue assay. The percentage of viable cells was calculated by normalizing the fluorescence intensity of 5-FC–treated wells to that of the corresponding untreated wells for each MSC proportion. Data are presented as means ± SD from triplicate experiments.

The tumor cell-killing effect of RRV-mediated suicide gene therapy was evaluated in a co-culture model using MSTO-211H/mCherry cells mixed with fully infected MSC-ad/RRV-CD cells at varying ratios (0–8%). Cells were cultured for 6 days in the presence of the 5-FC prodrug, and cytotoxicity was assessed. Tumor cell death was largely dependent on the MSC proportion and 5-FC concentration (Fig. 4). Notably, an MSC ratio exceeding 0.5% induced > 90% tumor cell death at a 5-FC concentration of 10 mM.

### In vivo RRV transmission from MSCs to peritoneally disseminated tumors

**Fig. 5:**
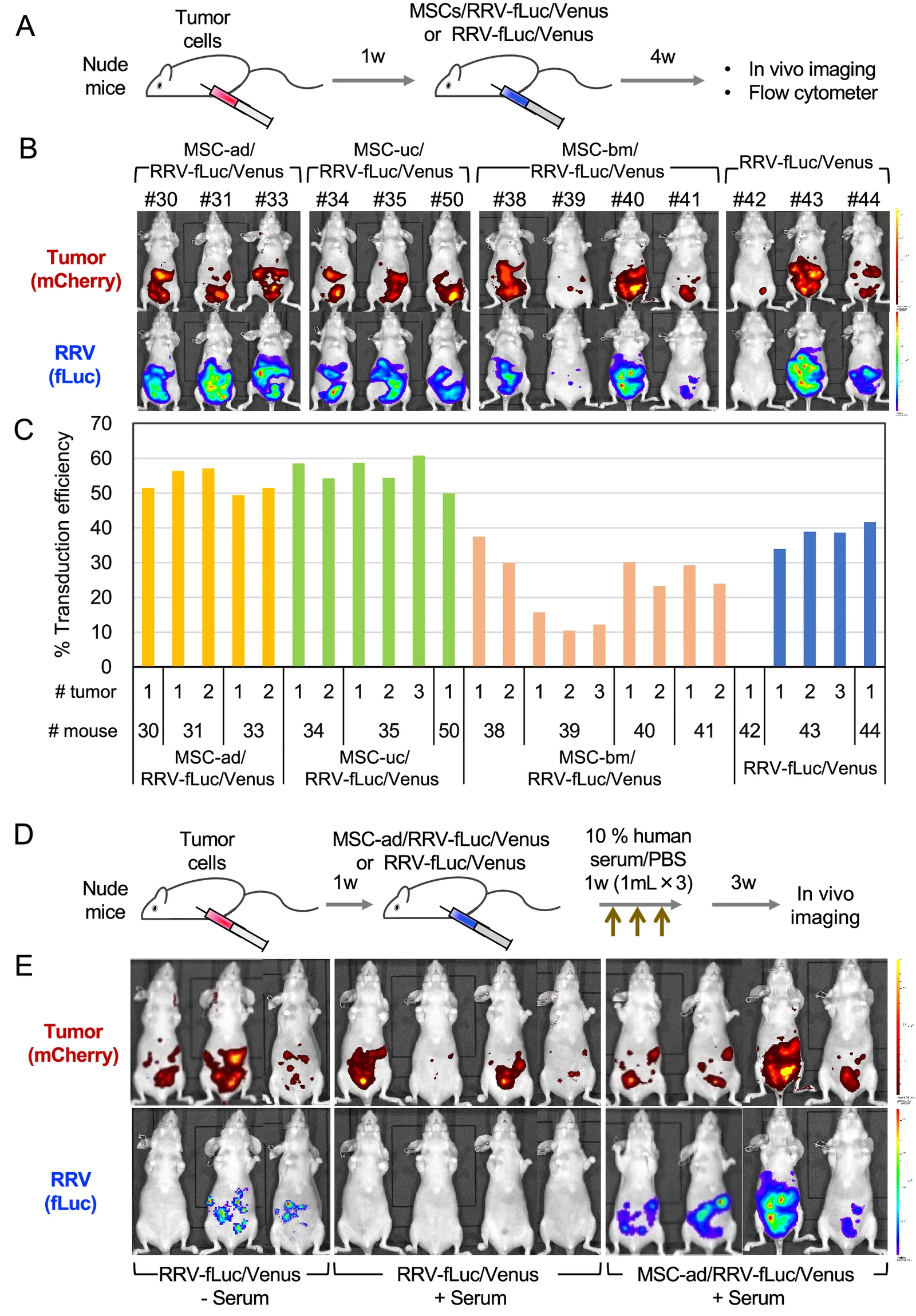
In vivo transmission of RRV from MSCs to peritoneally disseminated tumors. **A** Experimental outline of a mesothelioma xenograft model without ascites. To establish intraperitoneal tumors, nude mice were injected intraperitoneally with MSTO-211H/mCherry cells. One week later, RRV-fLuc/Venus or MSCs (MSC-ad, MSC-bm, MSC-uc) fully infected with RRV-fLuc/Venus were administered intraperitoneally. Four weeks later, mCherry and fLuc expression was analyzed using the IVIS system (**B**). Individual tumors were then harvested from the abdominal cavities of each mouse, dissociated into single-cell suspensions, and analyzed for Venus expression using flow cytometry (**C**). **B** In vivo imaging of tumor and RRV-infected cells in non-ascites models. Tumor cells are shown in red (mCherry-positive), and RRV-fLuc/Venus-infected cells are shown in blue (fLuc-positive). **C** Flow cytometric analysis of Venus-positive tumor cells. Tumors were harvested, digested into single-cell suspensions, and analyzed for Venus expression using flow cytometry. **D** Experimental outline of a mesothelioma xenograft model with ascites. Nude mice were injected intraperitoneally with MSTO-211H/mCherry cells to establish peritoneally disseminated tumor models. One week later, RRV-fLuc/Venus or MSC-ad transduced with RRV-fLuc/Venus (MSC-ad/ RRV-fLuc/Venus) was administered intraperitoneally. Certain groups also received intraperitoneal injections of 1 mL PBS containing 10% human serum three times (days 0, 2, and 4 after injection). Three weeks later, mCherry and fLuc expression was analyzed using the IVIS system. **E** In vivo imaging of tumor and RRV-infected cells in ascites models.

In vivo RRV transmission from MSCs to tumors was assessed using a peritoneal mesothelioma xenograft model. One week after intraperitoneal inoculation of MSTO-211H/mCherry tumor cells, fully infected MSCs (MSC-ad, MSC-bm, or MSC-uc) carrying RRV-fLuc/Venus were administered intraperitoneally (Fig. 5A). The control group received direct intraperitoneal administration of RRV-fLuc/Venus. All MSC subtypes successfully delivered RRVs to the tumors (Fig. 5B). Flow cytometric analysis of Venus expression in isolated tumor cells revealed that MSC-ad and MSC-uc achieved higher transduction efficiency than MSC-bm or direct RRV-fLuc/Venus administration (Fig. 5C). These results demonstrate that MSC-ad and MSC-uc efficiently transport RRVs to peritoneally disseminated tumors in vivo.

### Transmission of RRV in the presence of ascites

RRV transmission from MSCs to tumors was further evaluated in mice with ascites. As MSTO-211H cells do not naturally produce ascites, PBS containing human serum was used to mimic ascitic conditions (Fig. 5D). Under these conditions, direct intraperitoneal administration of RRV-fLuc/Venus resulted in markedly reduced tumor transduction efficiency (Fig. 5E). In contrast, MSC-mediated RRV delivery maintained efficient tumor transduction, underscoring the robustness of MSCs as a delivery vehicle under ascites-mimicking conditions.

### Therapeutic efficacy of MSC-mediated RRV suicide gene therapy

**Fig. 6:**
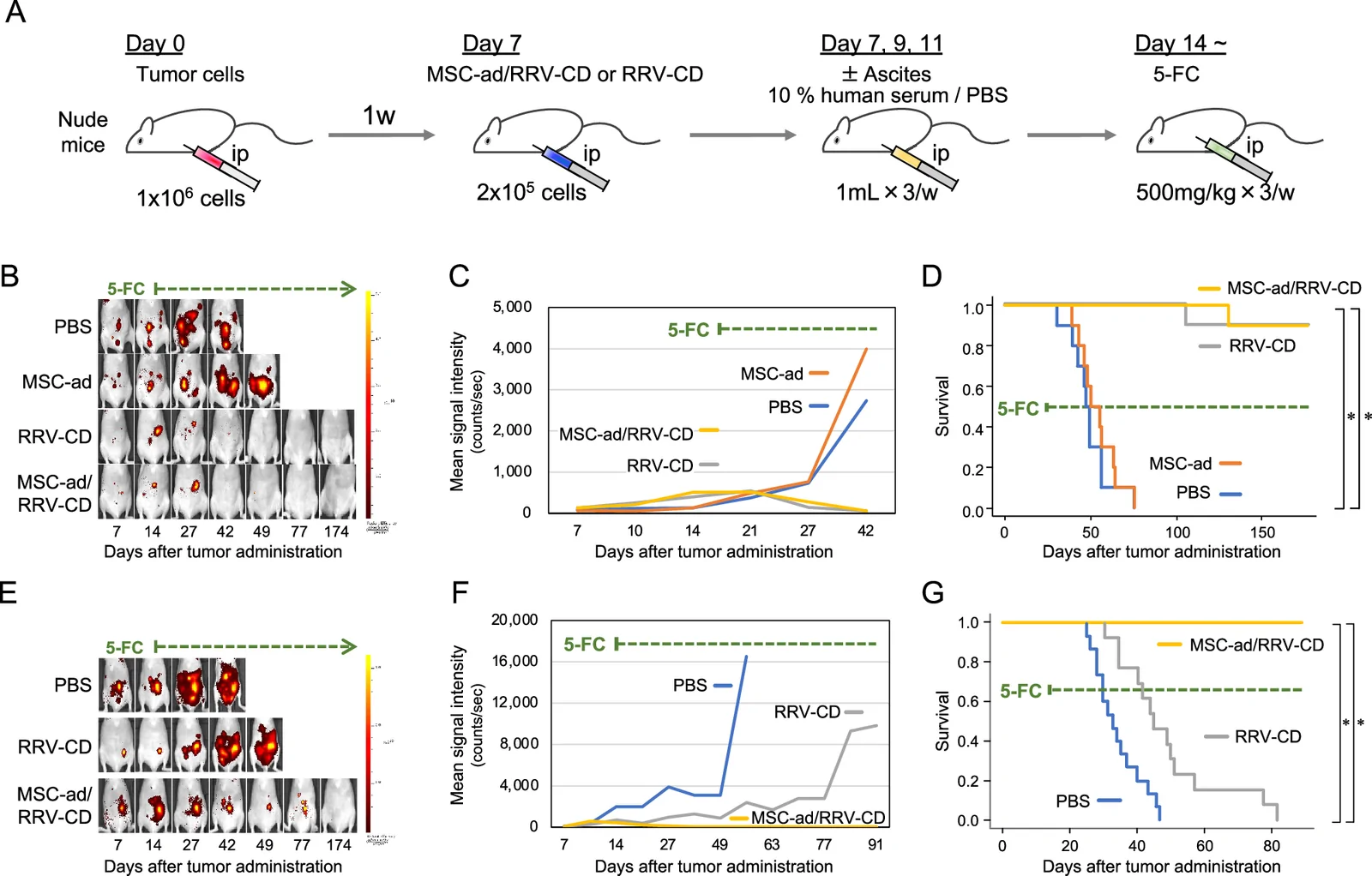
Therapeutic effects of RRV-mediated suicide gene therapy in a peritoneal mesothelioma xenograft model. **A** Experimental outline. MSTO-211H/mCherry tumors were established intraperitoneally in nude mice. Mice were treated with PBS, MSC-ad, RRV-CD, or MSC-ad/RRV-CD on day 7 (*n* = 10 per group). Mice in the ascites model received intraperitoneal injections of 1 mL PBS containing 10% human serum on days 7, 9, and 11. Starting on day 14, 5-FC was intraperitoneally administered three times per week until the end of the experiment. **B** Fluorescent imaging of tumors in non-ascites models. Representative images of each group. **C** Fluorescent signal intensities in non-ascites models. Quantification using the ROI analysis in the Living Image software. Data are presented as mean values for each group. **D** Survival analysis in the non-ascites models. Kaplan–Meier curves (*p < 0.01). **E** Fluorescent imaging of tumors in ascites models. Representative images of each group. **F** Fluorescent signal intensities of tumors in ascites models. Quantification using the ROI analysis. Data are presented as mean values for each group. **G** Survival analysis in ascites models. Kaplan–Meier curves (*p < 0.01).

The therapeutic efficacy of MSC-mediated RRV delivery was evaluated in a peritoneally disseminated tumor xenograft model under both ascitic and non-ascitic conditions. Nude mice bearing MSTO-211H/mCherry tumors (*n* = 10 per group) received a single intraperitoneal injection of PBS, MSC-ad, RRV-CD, or RRV-CD-carrying MSC-ad, followed by systemic 5-FC administration (Fig. 6A).

In the absence of ascites (Fig. 6B–D), intraperitoneal tumor fluorescence progressively increased in the PBS and MSC-ad groups, accompanied by emaciation, weakness, and impaired weight gain, with all mice succumbing by day 75. In contrast, tumor fluorescence was eliminated in the RRV-CD and MSC-ad/ RRV-CD groups (Fig. 6B, C), and all mice in these groups survived beyond day 75 (Fig. 6D). One mouse in each group died on days 105 and 130 in the RRV-CD and the MSC-ad/RRV-CD groups, respectively, whereas the remaining nine mice in both groups survived until day 174.

In the presence of ascites (Fig. 6E–G), tumor fluorescence continued to increase in the PBS and RRV-CD groups (Fig. 6E, F), with all mice dying by day 56 and day 98, respectively (Fig. 6G). In contrast, the MSC-ad /RRV-CD group showed significantly greater tumor suppression (Fig. 6E, F) and a marked survival benefit (Fig. 6G) than the RRV-CD group, without any observable body weight loss or decrease in general activity throughout the experimental period. These findings demonstrate that MSC-mediated RRV delivery effectively overcomes ascites-associated inhibition and achieves robust therapeutic efficacy against peritoneally disseminated tumors.

## Discussion

In this study, we investigated the feasibility and therapeutic utility of using MSCs as novel carriers for RRVs, focusing on a clinically relevant model of peritoneal mesothelioma with malignant ascites. We demonstrated that MSCs, particularly adipose-derived MSCs (MSC-ad), can be efficiently infected with RRVs, support viral production, and migrate toward tumor cells even under ascites-mimicking conditions. Importantly, MSC-based RRV suicide gene therapy produced marked antitumor effects, including significant tumor suppression and prolonged survival, and outperformed conventional direct RRV delivery approaches in vivo.

This strategy was designed to address the challenges of peritoneally disseminated cancers, such as peritoneal mesothelioma and ovarian or gastrointestinal carcinomas, which frequently coexist with cancerous ascites and remain refractory to current treatments. To reproduce this clinical condition, we developed a peritoneal dissemination model using human pleural mesothelioma-derived MSTO-211H cells and introduced 10% human serum to mimic the virus-inactivating properties of ascitic fluid. In this stringent setting, MSCs effectively transported and transmitted RRVs to tumors, overcoming the limitations of direct RRV administration. These findings support the potential of MSC-based RRV delivery as a clinically relevant strategy for treating ascites-complicated intraperitoneal malignancies. However, in the present study, therapeutic intervention was initiated concurrently with ascites induction, representing a prophylactic-like setting rather than treatment of fully established ascites. Accordingly, the impact of treatment timing relative to ascites development was not directly assessed. Moreover, human serum represents a simplified and partial surrogate for malignant ascites and does not fully reproduce its complex biological composition, which includes tumor- and host-derived cytokines, growth factors, extracellular vesicles, and cellular components. Therefore, further studies employing authentic tumor-associated ascites samples and more physiologically relevant ascites models will be required to rigorously validate the robustness and translational applicability of MSC-mediated RRV delivery.

Previous studies have examined MSCs as carriers for various oncolytic viruses, including those derived from measles virus [21], adenovirus [22–24], and herpesvirus [25], in tumor models such as ovarian cancer [21], hepatocellular carcinoma [24], malignant glioma [22, 25], and pleural disseminated carcinoma from bladder cancer [23]. For instance, MSC-mediated delivery of the oncolytic measles virus in an ovarian cancer model reduced the incidence of ascites to 20% and doubled survival [21]. Similarly, adenovirus-transfected MSCs prolonged survival by approximately three-fold in a pleural carcinoma model [23]. Although these strategies demonstrated temporary tumor suppression and survival benefits, the cytolytic and immunogenic nature of these viruses likely compromised MSC function and limited their therapeutic effect. In contrast, RRVs are non-cytolytic and minimally immunogenic, which likely preserved MSC viability, migratory ability, and tumor-targeting efficiency. These properties may explain the effective in vivo RRV delivery (Fig. 5) and the enhanced therapeutic outcomes observed in our intraperitoneal tumor model with ascites (Fig. 6).

Among the MSC types tested in this study, MSC-ad exhibited superior migratory capacity toward cancer cells. Although MSC-ad showed relatively low RRV infection efficiency (Fig. 2A), this limitation was mitigated by optimizing the culture conditions. Additionally, MSC-ad demonstrated relatively low RRV production efficiency (Fig. 2B), which may be advantageous from a safety perspective by reducing the risk of unintended infection of non-tumor cells. Notably, when MSCs were in direct contact with cancer cells, MSC-ad efficiently transmitted RRVs to cancer cells, indicating that RRV transmission within tumors is highly efficient (Fig. 3). Importantly, MSC-ad enabled efficient RRV transmission to a large number of tumors, even in the presence of human serum, which was used as a surrogate for ascites-associated viral inactivation (Fig. 5D, E). Nevertheless, because human serum does not fully recapitulate the complex biological composition of tumor-associated ascites, further studies employing authentic ascites samples and more physiologically relevant models will be required to confirm the robustness of MSC-mediated RRV delivery under clinically relevant conditions. Furthermore, MSC-ad exhibited high proliferative capacity and resistance to spontaneous differentiation [29–31], whereas MSC-bm exhibited reduced proliferative capacity and tended to undergo earlier differentiation [32, 33]. Collectively, these features make MSC-ad a highly suitable vehicle for intraperitoneal delivery of RRVs. However, given the widely recognized functional heterogeneity of MSCs depending on donor variability, tissue origin, and culture conditions [16], further validation using multiple independent donors and tumor models is necessary to determine whether MSC-ad can consistently serve as the optimal carrier for RRV-based therapies across diverse experimental and clinical settings.

The clinical potential of MSC-based RRV delivery extends beyond peritoneal mesothelioma to other peritoneally disseminated cancers. These malignancies are frequently complicated by cancerous ascites, a common and debilitating manifestation of advanced disease [34, 35]. An ideal therapeutic strategy for such cases would involve the intraperitoneal administration of anticancer viruses, enabling efficient and selective delivery to individual tumor nodules within the peritoneal cavity, even in the presence of ascites. In this context, MSC-mediated RRV delivery offers significant promise, combining robust antitumor efficacy with minimal off-target effects on normal tissues. Additional studies using diverse cancer models will be essential to validate and expand upon this promising therapeutic approach.

Moreover, MSCs may provide additional therapeutic benefits by addressing cancerous ascites, a severe complication that causes abdominal distension, pain, nausea, and shortness of breath, significantly impairing patients’ quality of life. Ascites development is multifactorial and is driven by tumor-secreted factors, such as vascular endothelial growth factor (VEGF) and hepatocyte growth factor (HGF), which increase peritoneal angiogenesis and vascular permeability, as well as by tumor-induced lymphatic obstruction [36]. MSCs are known for their ability to promote tissue repair and modulate inflammatory processes [37–39] and exhibit natural tumor-homing properties, making them potential candidates for targeting the pathological mechanisms underlying ascites production [37]. By regulating angiogenesis and inflammation, MSCs may contribute to suppression of ascites development, thereby improving both clinical outcomes and patient quality of life.

The impact of MSCs on tumor growth requires further investigation. Although MSCs can promote tumor progression by secreting growth factors (such as HGF) and cytokines (such as IL-6) [40, 41], they also inhibit cancer proliferation by inducing apoptosis and arresting the cell cycle [42]. In this study, MSC-ad did not promote tumor growth in the MSTO-211H model, indicating a minimal risk of adverse effects in this context (Fig. 6B–D). However, the influence of MSCs on cancer progression may vary according to tumor type and should be carefully evaluated.

For future clinical application, the use of allogeneic, commercially available MSCs is a practical approach. These cells are expected to remain viable in the body for a limited period (approximately a few days) [43–46], thereby minimizing their potential impact on tumor progression while allowing sufficient time for effective RRV delivery. Additionally, the bystander effect of RRV suicide gene therapy can efficiently induce cell death in both tumor cells and MSCs within the cancer microenvironment, further enhancing safety and efficacy [1]. Therefore, from both safety and efficacy perspectives, MSC-based RRV delivery may be an excellent therapeutic option for many cancers. Nevertheless, although extensive preclinical and clinical data support the safety of RRV-based suicide gene therapy, further evaluation of the interactions between MSC-ad/RRV and normal cells or tissues may provide additional mechanistic insights and warrants investigation in future studies.

In conclusion, this study provides strong preclinical evidence that MSC-based RRV suicide gene therapy represents a promising and clinically relevant strategy for treating peritoneally disseminated cancers with ascites. This approach could substantially expand the therapeutic applicability of RRV gene therapy and improve outcomes for patients with metastatic malignancies by leveraging the tumor-homing and immunologically inert properties of MSCs.

## Data Availability

The data presented in this study are available from the corresponding author upon request.
